# Genetic diversity analysis of two commercial breeds of pigs using genomic and pedigree data

**DOI:** 10.1186/s12711-016-0203-3

**Published:** 2016-03-30

**Authors:** Ricardo Zanella, Jane O. Peixoto, Fernando F. Cardoso, Leandro L. Cardoso, Patrícia Biegelmeyer, Maurício E. Cantão, Antonio Otaviano, Marcelo S. Freitas, Alexandre R. Caetano, Mônica C. Ledur

**Affiliations:** Embrapa Swine and Poultry National Research Center, Animal Breeding and Genetics, Concordia, SC Brazil; Embrapa Southern Region Animal Husbandry, Bagé, RS Brazil; BRF/SA, Curitiba, PR Brazil; Programa de pós-graduação em Zootecnia/UFPel, Pelotas, RS Brazil; Embrapa Recursos Genéticos e Biotecnologia, Brasília, DF Brazil; Faculdade de Agronomia e Medicina Veterinária (FAMV), University of Passo Fundo, Passo Fundo, RS Brazil; Programa de pós-graduação em Ciências Animais/Universidade de Brasília, Brasília, DF Brazil; Programa de pós-graduação em Zootecnia/Campus UDESC Oeste, Universidade do Estado de Santa Catarina, Chapecó, SC Brazil

## Abstract

**Background:**

Genetic improvement in livestock populations can be achieved without significantly affecting genetic diversity if mating systems and selection decisions take genetic relationships among individuals into consideration. The objective of this study was to examine the genetic diversity of two commercial breeds of pigs. Genotypes from 1168 Landrace (LA) and 1094 Large White (LW) animals from a commercial breeding program in Brazil were obtained using the Illumina PorcineSNP60 Beadchip. Inbreeding estimates based on pedigree (*F*_x_) and genomic information using runs of homozygosity (*F*_ROH_) and the single nucleotide polymorphisms (SNP) by SNP inbreeding coefficient (*F*_SNP_) were obtained. Linkage disequilibrium (LD), correlation of linkage phase (*r*) and effective population size (*N*_*e*_) were also estimated.

**Results:**

Estimates of inbreeding obtained with pedigree information were lower than those obtained with genomic data in both breeds. We observed that the extent of LD was slightly larger at shorter distances between SNPs in the LW population than in the LA population, which indicates that the LW population was derived from a smaller *N*_*e*_. Estimates of *N*_*e*_ based on genomic data were equal to 53 and 40 for the current populations of LA and LW, respectively. The correlation of linkage phase between the two breeds was equal to 0.77 at distances up to 50 kb, which suggests that genome-wide association and selection should be performed within breed. Although selection intensities have been stronger in the LA breed than in the LW breed, levels of genomic and pedigree inbreeding were lower for the LA than for the LW breed.

**Conclusions:**

The use of genomic data to evaluate population diversity in livestock animals can provide new and more precise insights about the effects of intense selection for production traits. Resulting information and knowledge can be used to effectively increase response to selection by appropriately managing the rate of inbreeding, minimizing negative effects of inbreeding depression and therefore maintaining desirable levels of genetic diversity.

## Background

Pork is a low-cost source of high-quality animal protein and is produced for human consumption worldwide. Pork production is very important for the economy of many countries from the European Union, China, USA and Brazil, which ranks as the fourth largest pork producer in the world, with 3.39 million metric tons of meat produced per year [1]. Two breeds are mainly used as maternal lines in Brazilian swine breeding programs: Landrace (LA) and Large White (LW), which represent 20.2 and 18.9 % of the overall germplasm used for pork production, respectively [1]. These two breeds alone were responsible for approximately 40 % of all animals registered in the National Swine Producers Association until 2013 [1]. However, little is known about the current levels of genetic diversity within these two breeds in the Brazilian swine herd.

Intense animal selection and different mating strategies have been used to improve production traits within the livestock sector [2]. Although higher selection intensities can lead to faster genetic progress [3], undesirable increases in inbreeding levels may result as a consequence. In addition, overuse and misuse of assisted reproductive technologies such as artificial insemination may exacerbate these effects [4], since the excessive use of specific sires to improve specific traits may lead to undesirable losses of genetic diversity. High levels of inbreeding lead to the accumulation of high levels of homozygosity in animals within a herd, which in turn is likely to have detrimental consequences on traits that are related to reproduction, body conformation, growth and immune response [5].

Recent technological advances in methods to generate genome-wide sequencing and genotyping data have significantly improved the well-established processes for pedigree testing and confirmation of paternity assignments [6]. High-density genomic data have also been successfully used to identify quantitative trait loci (QTL) that affect health, behavioral and production traits in several species, including pigs [7–10]. More recently, high-density single nucleotide polymorphism (SNP) panels have also been used to estimate genetic diversity parameters for breeds or lines [11, 12].

The use of genomic information for pedigree correction and/or breeding and selection [13] can greatly improve accuracy of estimated breeding values and reduce generation intervals via the genomic evaluation of young animals before phenotypes can be measured. Therefore, significant increases in the rate of genetic gain are expected and have been reported in cattle [14]. However, at the same time, inbreeding can also increase at a higher rate if it is not properly considered in the selection and mating strategies [15, 16].

Inbreeding levels are conventionally estimated with pedigree information and depend strongly on the accuracy and amount of pedigree data [15, 17]. Better accuracies can be obtained when inbreeding estimates are derived from genome-wide SNP data [18]. However, since alleles at a locus that are identical by descent (IBD) versus identical by state (IBS) cannot be distinguished, these methods can result in overestimated inbreeding levels compared to pedigree-based estimates [19]. An alternate approach to control these issues is to use estimates that are obtained from observed runs of homozygosity (ROH). Runs of homozygosity are defined as contiguous stretches of homozygous genotypes that are present in an animal due to both parents transmitting identical haplotypes to their offspring. ROH provide a more accurate prediction of alleles at a locus that are IBD, and have been widely used in studies on human populations to accurately estimate levels of autozygosity among individuals [20].

High levels of homozygosity are generally associated with the segregation of long stretches of homozygous regions across the genome, which in turn increases the chance that deleterious alleles are expressed in populations. In addition, increased levels of homozygosity are associated with reduced effective population size (*N*_*e*_), which is a measure of within-breed diversity that describes the inbreeding rate by generation and the loss of genetic variation [21]. It has been shown that the use of high-density SNP panels improves the accuracy of population parameter estimates, such as *N*_*e*_, inbreeding across generations, linkage disequilibrium (LD) between loci within a population, and correlation of linkage phase, which is a measure of the degree of agreement of linkage phase for pairs of SNPs between two populations [17]. These parameters are useful measures of similarity within and across breeds [17]. Therefore, the objectives of this study were to investigate the within- and between-population diversity of two maternal purebred lines of distinct porcine breeds (LA and LW) in a commercial breeding program in Brazil, using pedigree and genomic data. In addition, conserved regions based on shared ROH within and between lines were further characterized and explored.

## Methods

This study was conducted at Embrapa Swine and Poultry National Research Center and was approved by the Institutional Ethics Committee on Animal Utilization for all experimental protocols used.

### Animal populations

All samples used in this study were obtained from two maternal lines of pigs from a commercial breeding nucleus (BRF/SA, Curitiba, PR, Brazil). A total of 1178 LA and 1200 LW DNA samples were genotyped. Animals were chosen according to their availability in the herd, while maximizing the number of breeders chosen. LA animals were born between 2006 and 2011 and LW animals between 2007 and 2011. For all the animals, complete pedigree records were available with an average depth of 6.4 and 5.6 generations for LA and LW, respectively, and information on a total of 84,611 LA and 50,348 LW animals in the pedigree.

### Sample collection and genotyping

DNA extraction was performed on 200 mg of frozen tissue from 2378 animals using the PureLink^®^ Genomic DNA Mini Kit (Invitrogen, San Diego, CA), according to the manufacturer’s instructions. Quantity and quality of DNA were measured with a NanoDrop ND-2000 spectrophotometer (NanoDrop Technologies Inc., Wilmington, DE). The 260/280 nm readings for all samples ranged from 1.8 to 2.0. Samples were diluted to a final concentration of 500 ng and genotyped by a commercial lab (GeneSeek, Lincoln, Nebraska, USA) using the Illumina PorcineSNP60 V2 BeadChip.

### Animal and SNP quality control

Prior to analysis of the genotyping data, 116 animals were excluded from the dataset based on the following criteria and using PLINK [22]: call rate less than 0.90, a level of heterozygosity higher than 3 standard deviations from the mean, and duplicated samples (match level >99 %). Pedigree errors based on IBD levels (sire or dam to offspring and full-sibs IBD ~0.5, half-sibs IBD ~0.25 and first cousins IBD ~0.125) and sex mis-assignments based on X chromosome inbreeding estimates (F) using standard values of F < 0.2 and >0.8 for females and males, respectively, were also verified using PLINK [22].

For the analysis of genomic inbreeding, SNPs with an unknown position based on the Pig60K_SNP_pos_build 10.2 (see http://www.animalgenome.org/repository/), SNPs with a call rate higher than 0.90, and SNPs located on sex chromosomes were removed. A total of 45,766 SNPs were used to estimate genomic inbreeding in the LA and LW pig breeds.

Additional data pruning was performed with R snpStats (v 1.14.0) to prepare data for analyses of LD, correlation of linkage phase, and *N*_*e*_ [23]. The following quality control (QC) criteria were used to remove SNPs that had a call rate lower than 0.98, a minor allele frequency (MAF) lower than 0.03 and that deviated significantly from Hardy–Weinberg equilibrium (p < 10^−6^). The final dataset contained 41,041 SNPs for LA and 36,452 SNPs for LW, and a total of 2262 samples, i.e. 1168 for LA (91 males and 1077 females) and 1094 for LW (114 males and 980 females). Sporadically missing genotypes were imputed during the phasing procedure using FImpute software [24].

Quality control was performed on both breeds for genomic inbreeding estimates, while for estimates of *N*_*e*_, LD and correlation of linkage phase, it was performed within breed to avoid SNPs being penalized by the HWE criterion, since some SNPs can be fixed within one breed only.

### Linkage disequilibrium, correlation of linkage phase and effective population size

Pairwise linkage disequilibrium (*r*^*2*^) estimates [25] were calculated using ld_estimate R scripts [26]. For each breed, LD values between all pairs of SNPs within each chromosome were grouped according to their pairwise physical distance in classes of 100 kb, starting from 0 to 10 Mb. Summarized *r*^2^ values at different distances were obtained by calculating the mean across all chromosomes. In addition, *r*^2^ estimates were also calculated using only one in every 10 SNPs to mimic a lower density SNP panel.

The correlation of linkage phase (*r*) for SNP pairs between the two breeds was calculated following Badke et al. [26], using the same grouping strategy as applied for *r*^2^. The mean values of *r* according to distances between SNPs were calculated using the SNPs that were shared by both breeds.

The relationship between *r*^*2*^ and *N*_*e*_ was calculated following [27]:

$$E\left( {r^{2} } \right) = 1/\left( {4cN_{e} + 1} \right),$$

where *c* is the genetic distance between two SNPs expressed in Morgan. Based on this equation, *N*_*e*_ for *t* generations in the past (*N*_*et*_) can be estimated using the relationship between *t* and *c* (*t* = 1/2*c*) [28] and solving *N*_*e*_ as:

$$N_{et} = \left( {1 - r^{2} } \right)/\left( {4cr^{2} } \right), \quad \hbox{for} \, 0.0 < r^{2} < 1.0.$$

First, physical distances between SNPs within each chromosome were converted to genetic distances considering 1 cM ~ 1 Mb [17]. Because generations were assumed to be discrete and distances between SNPs are continuous, to calculate *N*_*et*_, estimates of *r*^2^ for a range of values of *c* were used which, when applied in *t* = 1/2*c*, rounded to the target generation. For example, *r*^2^ of all SNP pairs with distances between 0.333 (*t* = 1.5) and 1 M (*t* = 0.5) were selected and averaged across all chromosomes to calculate *N*_*e*_ at *t* = 1. Due to the inverse relationship between *t* and *c*, as *t* increased, wider intervals around *t* were used to define the corresponding ranges of* c* to ensure that sufficient numbers of SNP pairs were used to reliably estimate *N*_*et*_ for each *t* [27]. Values of *N*_*et*_ were obtained with increments of one generation for *t* = 1, 2,…, 10, of five generations for *t* = 15, 20 …, 100, and of 50 generations for *t* = 150, 200,…, 1000 [29].

### Inbreeding estimates based on pedigree and genotypic data

Pedigree-based inbreeding (*F*_x_) was estimated according to Wright’s coefficient [30] with the R pedigree package [31] for each population as a whole. Runs of homozygosity were calculated with PLINK [22], using the following parameters: a minimum ROH of 50 SNPs with a minimum length of 1000 kb, and one heterozygous SNP and one missing SNP genotype were allowed within a sliding window of 50 SNPs [4, 11]. Identified ROH were then used to estimate individual genomic inbreeding coefficients (*F*_ROH_) following [4]:

$$F_{\text{ROH}} = \frac{{\mathop \sum \nolimits_{k} {\text{Length}}\left( {{\text{ROH}}_{k} } \right)}}{\text{L}},$$

‬where *k* is the number of ROH identified for the individual, multiplied by the average length of its ROH segments, and L is the total swine genome length (2,808,525 kb, Sscrofa10.2, Aug 2011). Genomic SNP-by-SNP inbreeding coefficients (*F*_SNP_) were obtained based on the proportion of homozygous genotypes for each individual, which measures the observed percentage of homozygosity per animal. The three methods of estimating inbreeding were then compared within-breed using Pearson correlations.

### Identification of conserved regions and gene content in shared homozygous regions

A comparison of the percentage and number of shared ROH among individuals was performed with a Pearl homemade script to identify conserved regions within and between breeds. For the identification of shared ROH within breeds, shared identical segments observed in different animals of the same breed were considered. To define shared ROH between breeds, an identical segment with the same start and end points found between animals of the different breeds was used. Further analyses were carried out using the UCSC genome browser [32], to search for positional candidate genes located on the identified shared ROH.

## Results

### Linkage disequilibrium, correlation of linkage phase and effective population size

**Fig. 1:**
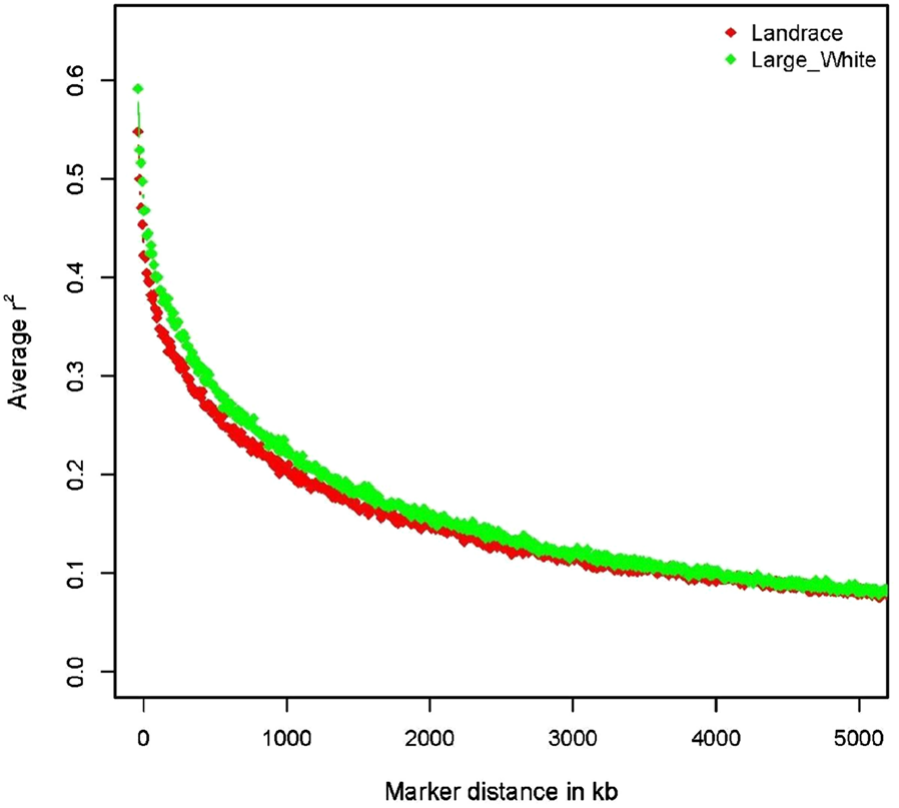
Decay of average pairwise linkage disequilibrium (*r* ^2^) over distance between SNPs in Landrace and Large White populations

Results for LD between adjacent markers (*r*^2^) and persistence of linkage phase (*r*) are in Table 1. Average *r*^2^ for adjacent SNPs was slightly higher for the LW (*r*^2^ = 0.50) than for the LA breed (*r*^2^ = 0.46). Figure 1 shows the average *r*^2^ by distance between SNPs in classes of 100 kb, which provides an overview of the decline of *r*^2^ over distance in each breed. At shorter distances, the average *r*^2^ was higher in LW than in LA. Breed differences decreased as distance between markers increased and approached background LD levels at 5 Mb distances in both breeds, with average *r*^*2*^ ≈ 0.08. Average *r*^2^ values for neighboring SNPs calculated by using one in every 10 available SNPs were equal to 0.27 for LA and 0.30 for LW.

**Table 1:** Inbreeding coefficient estimates, linkage disequilibrium and correlations of linkage phase for Landrace and Large White populations

	Landrace	Large White
*F*_x_	0.014 (0.0003)	0.021 (0.0003)
*F*_ROH_	0.094 (0.0006)	0.106 (0.0007)
*F*_SNP_	0.668 (0.0005)	0.667 (0.0512)
*r* ^2^	0.459 (0.002)	0.497 (0.002)
*r*	0.770	

Numbers presented are the average and its standard error; *F*_x_ = pedigree-based inbreeding coefficient; *F*_ROH_ = genomic inbreeding based on runs of homozygosity (ROH); *F*_SNP_ = genomic inbreeding based on the proportion of SNPs that are homozygous (SNP-by-SNP); *r* ^2^ = linkage disequilibrium among adjacent SNPs; *r* = correlations of linkage phase

**Fig. 2:**
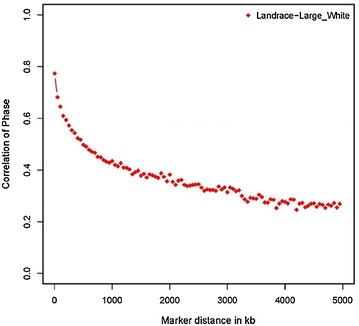
Correlation of linkage phase between breeds for SNP pairs grouped by distance in 100 kb intervals across the genome

The average distance between consecutive SNPs across all chromosomes was equal to 65.7 and 73.9 kb for LW and LA, respectively. For LA, 61 % of adjacent informative SNP pairs had *r*^2^ values higher than 0.2 and 53 % had *r*^2^ values higher than 0.3. For LW, informative SNPs that showed a level of LD higher than 0.2 and 0.3 represented 64 and 56 % of the adjacent SNP pairs analyzed, respectively. Correlations of linkage phase between the two breeds were moderately high (*r* = 0.77) between SNPs at distances up to 50 kb (Fig. 2).

**Fig. 3:**
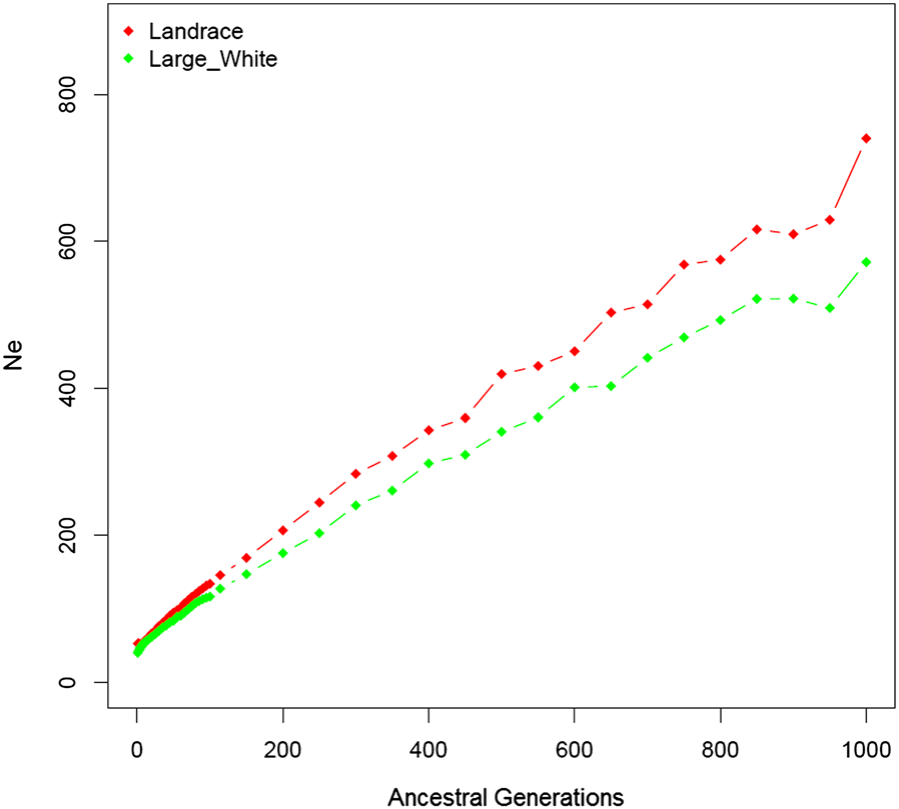
Effective population size (*N*_*e*_) of the Landrace and Large White populations

Estimates of *N*_*e*_ obtained for the past 1000 generations for LA and LW are in Fig. 3. Estimated *N*_*e*_ at 1000 generations back was equal to 572 for LW and 740 for LA. In the last five generations, *N*_*e*_ ranged from 50 to 53 for LA and from 40 to 48 for LW.

### Inbreeding coefficient estimates based on pedigree and genomic data

**Fig. 4:**
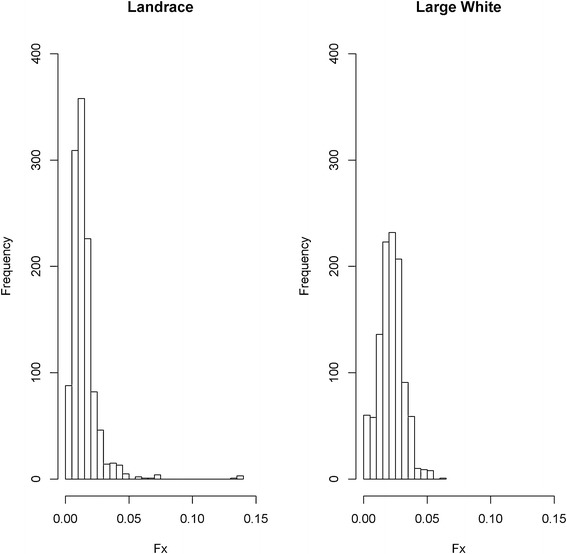
Inbreeding estimates based on pedigree information (*F*_x_)

Inbreeding coefficients estimated based on pedigree and genomic data for both breeds are in Table 1. Inbreeding estimates (*F*_x_) obtained by using all pedigree data available for both breeds ranged from 0 to 0.139 with an average of 0.014 for LA, and from 0 to 0.062 with an average of 0.021 for LW (Fig. 4). Although higher inbreeding levels were observed for some LA animals compared to the LW animals, the average *F*_x_ was higher for the LW than the LA breed.

**Fig. 5:**
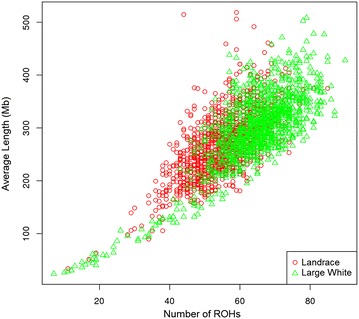
Relationship between the number of ROH and the average length of each ROH for the Landrace and Large White populations. Each point represents the number and average length of ROH of one animal. *Red circles* represent Landrace and *green triangles* represent Large White animals

The average number of observed ROH per genome was equal to 52.7 (ranging from 10 to 84) for LA, and 61.4 (ranging from 7 to 87) for LW. The size of the ROH ranged from 28.8 to 513.0 Mb (mean = 252.9 Mb) for LA and from 23.8 to 498.5 Mb (mean = 280.1 Mb) for LW (Fig. 5). Both the mean number and size of ROH differed significantly between LA and LW (p < 2.2^e−16^). The correlation between number and size of ROH within breed was 0.64 for LA and 0.78 for LW. Average genomic inbreeding based on observed ROH (*F*_ROH_) was equal to 0.09 (ranging from 0.0001 to 0.180) for LA and 0.10 (ranging from 0.008 to 0.177) for LW. Estimated *F*_SNP_ inbreeding coefficients ranged from 0.612 to 0.727 for LA (mean = 0.668) and from 0.611 to 0.717 (mean = 0.667) for LW.

Correlations between estimated levels of *F*_ROH_ and *F*_SNP_ were moderately high for both LA (*r*^*2*^ = 0.82) and LW (*r*^2^ = 0.71). Conversely, correlations between *F*_x_ and *F*_ROH_ and *F*_x_ and *F*_SNP_ were low for both LA (*r*^2^ = 0.24 and 0.21, respectively) and LW (*r*^2^ = 0.015 and 0.140, respectively).

### ROH, conserved regions, gene content and shared homozygosity

A region on SSC1 (SSC for *Sus scrofa* chromosome) that spanned 2.42 Mb (between 190,770,055 and 193,195,907 bp) was shared by 61.5 % of the LA individuals. A similar region was also shared among 56 % of the LW individuals (between 189,577,254 and 192,117,017 bp). This region did not contain any known annotated genes in the pig genome.

SSC14 contained the largest number of ROH for the LA breed. The region between 99,956,031 and 101,787,411 bp on SSC14 was shared among 629 LA individuals (53.9 %) and harbors two genes: *CXCL12* (*chemokine C*-*X*-*C motif ligand* 12) and *TFAM* (*transcription factor A mitochondrial precursor*). *CXCL12* was shown to be associated with immunological traits in the LA breed, especially with disease resistance, and may therefore be important for survival [33]. *TFAM* plays an important role in porcine gametogenesis and embryo preimplantation and development and thus may have broad implications in cell physiology and evolutionary biology [34]. A region on SSC4 (between 96,423,033 and 98,660,311 bp) was shared among 62 % of LW individuals and harbors 13 known genes with different functions.

A total of 1333 homozygous genomic regions were shared between the LA and LW breeds (Table 2), which suggests that they harbor important genes that have been under selection during domestication and contemporary breeding for production performance.

**Table 2:** Number of shared runs of homozygosity per chromosome between the Landrace and Large White populations

Chromosome	Number of shared regions	Number of SNPs per chromosome
SSC1	171	6378
SSC2	94	2645
SSC3	82	2201
SSC4	77	3504
SSC5	63	2168
SSC6	51	2118
SSC7	72	3347
SSC8	65	2223
SSC9	95	2615
SSC10	52	1347
SSC11	55	1769
SSC12	29	1123
SSC13	83	3562
SSC14	89	3866
SSC15	89	2609
SSC16	50	1553
SSC17	63	1609
SSC18	53	1129
Total	1333	45,766

## Discussion

The genetic diversity within and between two maternal breeds of pigs (LA—Landrace and LW—Large White) used in a commercial breeding nucleus was analyzed using pedigree and genomic information. Three methods were computed to estimate inbreeding coefficients: Wright’s pedigree-based inbreeding coefficient (*F*_x_), ROH (*F*_ROH_) and the SNP-by-SNP coefficient (*F*_SNP_). In addition, estimates of LD, *N*_*e*_ and correlation of linkage phase were calculated for both breeds, which are important parameters to appropriately manage the population’s genetic diversity, which is a condition to effectively increase long-term response to selection.

Our results revealed a larger *N*_*e*_ and lower levels of homozygosity for LA than for LW. Moreover, the larger size and larger number of ROH found for the LW breed suggest that recent consanguinity events occurred in this breed, which is congruent with the observed estimates of *N*_*e*_, inbreeding and LD. However, since quality control for the estimation of *N*_*e*_ and LD was carried out within breed, the number of and distance between the SNPs used were different for LA and LW. Therefore, these results should be taken with caution.

Previous evaluations of LD and *N*_*e*_ in Finnish Landrace and Yorkshire pigs using the Illumina PorcineSNP60 Beadchip generated average estimates of *r*^2^ between adjacent SNPs of 0.43 and 0.46, respectively [17], while Badke et al. [26] reported *r*^2^ estimates of 0.46, 0.44, 0.36 and 0.39 for Duroc, Hampshire, Landrace and Yorkshire breeds, respectively. These values are comparable to the average *r*^2^ values observed for LW (0.50) and LA (0.46) in our study.

Slightly larger LD estimates were observed at shorter distances for LW than for LA (Fig. 1), which suggests that the LW line was derived from a population that had a smaller ancient *N*_*e*_ (*N*_*e*1000_ = 572) than the LA breed (*N*_*e*1000_ = 740). Low *N*_*e*_ in recent generations (Fig. 3) has led to the fixation of some LD blocks in LA and LW. Some of these LD blocks overlapped, as was observed in the shared ROH between the two breeds (Table 2). Average *r*^2^ between adjacent SNPs that were calculated by using only one in every 10 available SNPs were equal to 0.27 and 0.30 for LA and LW, respectively. According to Meuwissen et al. [35], the average *r*^2^ between adjacent SNPs needs to be greater than 0.2 for genomic selection (GS) to be effective. Thus, within the LA and LW populations evaluated here, LD is present at useful levels (i.e. *r*^2^ > 0.3) [36, 37] and SNP panels with sparser densities could be used for genome-wide association studies (GWAS) and GS. This is in agreement with Badke et al. [26] and Veroneze et al. [38], who reported that LD levels extend further in the European swine genome than in the bovine genome, which implies that less dense SNP panels can be used to conduct GWAS and GS in pigs. Low-density custom SNP panels could represent a cost-effective alternative for commercial breeding programs that aim at incorporating genomic tools in routine testing, while having little impact on the accuracy of GS.

Correlation of linkage phase (*r*), which can be used to infer the history of a species and relatedness of breeds within species [12, 39], showed moderate values for LA and LW. For SNPs that were up to 50 kb apart, the average *r* was equal to 0.77. However, *r* dropped sharply as the distance between SNPs increased, which indicates that estimation of SNP effects on performance traits of LA pigs based on SNP effects derived from LW pigs, and vice versa, could only be accurate if denser SNP panels were used, with distances between SNPs less than 5 kb. Similar results were found for North American purebred pig populations, with pairwise *r* at 10–50 kb between the Duroc, Hampshire, Landrace and Yorkshire populations ranging from 0.76 to 0.84 [26]. Our results indicate that the average gametic phase of two SNPs that are in LD is similar in both breeds analyzed. However, the phase correlations for pairs of SNPs that we observed between the LA and LW populations with the Illumina PorcineSNP60 Beadchip, limit the accuracy of inferring linkage phase in one breed based on estimates obtained from the other breed. To perform across-breed genomic prediction, *r* values higher than 0.8 are needed for SNP effects to remain consistent across breeds [14, 39] and, thus, denser SNP panels are required for these populations. Hence, GS based on the Illumina PorcineSNP60 Beadchip must be performed within breed.

In pigs, selection is carried out on pure lines to improve performance of crossbred animals, which imposes a challenge to the implementation of GS since phenotypes of interest are measured on crossbred animals and for a small number of pure lines. Veroneze et al. [40] reported low levels of accuracy for GS when using across-population data in pigs, possibly due to the low relationship among breeds with different LD levels.

Chromosomal segments that contain homozygous SNP genotypes can be used to infer possible haplotypes that are inherited by the same individual, and subsequently estimate genomic inbreeding coefficients using ROH (*F*_ROH_) [41]. Furthermore, ROH length is negatively correlated with time of coancestry, since long ROH are observed when recent consanguinity events occur within a pedigree. In contrast, shorter ROH are produced by IBD genomic regions from old ancestors and are indicative of more ancient relatedness, which is frequently unaccounted for in an individual’s recorded pedigree [12]. On average, longer stretches of ROH were observed for LW (~280 Mb) than for LA (~252 Mb) (p < 2.2^e−16^), which could be due to differences in the number of generations and selection strategy used by the company, with different levels of selection pressure on the traits to which these lines have been subjected to. These results are in agreement with the *N*_*e*_ estimates that were obtained for the current generation of each breed, with the LA population having a larger *N*_*e*_ than the LW population.

Inbreeding levels for the base LA and LW populations based on the available pedigree are unknown, thus levels of inbreeding based on *F*_x_ were expected to be lower than levels of inbreeding based on *F*_ROH_ and *F*_SNP_. Correlations between *F*_x_ and *F*_ROH_ estimates were low for both the LA (0.24) and LW (0.015) breeds. These results are in agreement with previous reports on populations of Wagyu cattle [4], Iberian pigs [19], and Duroc, Large White and Pietrain commercial pig lines [42]. Pedigree-based estimates assume that there is no inbreeding in the base population and thus this contributes to the low correlation. In addition, the use of genomic information allows Mendelian sampling effects to be estimated more accurately and thus improves the estimates of inbreeding rate [43]. The SNP-by-SNP inbreeding coefficient measures the increase in frequency of homozygous genotypes, including both IBD and IBS alleles (Table 1). Thus, *F*_*S*NP_ can overestimate the levels of inbreeding compared to *F*_ROH_ and *F*_x_. The correlation between the genomic inbreeding estimates using *F*_ROH_ and *F*_SNP_ were high in our study (*r*^2^ = 0.7), which is in agreement with previous results [18, 19].

Some studies have reported weak correlations between inbreeding estimates obtained from pedigree and genotype data in several species, such as Wagyu cattle [4], Duroc, Large White and Pietrain commercial pig lines [42], sheep and birds [44]. Lopes et al. [42] identified a low correlation (0.27) between inbreeding estimates based on SNP data versus pedigree data for a LW population. This correlation was higher than that found in our study between *F*_x_ and *F*_ROH_ for the LW population (*r*^2^ = 0.015). However, Lopes et al. [42] used a different methodology based on kinship and a considerably smaller number of SNPs, i.e. 28,740 compared to 47,069 SNPs in our study. In addition to differences in quality control thresholds, the difference in these correlations could also be due to distinct genetic backgrounds in these two LW populations. However, other studies have also reported high correlations between pedigree and genomic inbreeding estimates [6, 18, 19]. The high correlation found in [18, 19] can be partially explained by the low *N*_*e*_ of their populations (*N*_*e*_ = 10), which results in high levels of inbreeding (as in [18] i.e. *F*_x_ = 0.35 and *F*_SNP_ = 0.8). In this small Iberian pig population that has a low *N*_*e*_, *F*_x_ was possibly sufficiently accurate, especially because complete pedigree information was available. In this situation, genomic information may not add much to the inbreeding estimates, which results in a high correlation between genomic and pedigree-based inbreeding estimates. In our study, *N*_*e*_ in the last five generations varied between 40 and 53 in both populations and rigorous inbreeding control was applied. Therefore, low levels of inbreeding were observed and genomic inbreeding estimates were expected to be closer to the real inbreeding level than *F*_x_, which explains the low correlations found in our study. Saura et al. [19] reported a low negative correlation between *F*_x_ and *F*_ROH_Short_ (*r*^2^ = −0.24) in populations with high levels of pedigree-based inbreeding (*F*_x_ = 0.39) and SNP-by-SNP inbreeding (*F*_SNP_ = 0.86). This negative correlation was explained by the fact that their *F*_ROH_ estimates were based only on short segments of ROH (0.5 to 5 Mb), which mostly account for old inbreeding events. When long ROH segments (>5 Mb) were included in the analysis, the correlation between *F*_x_ and *F*_ROH_ improved and reached a value of 0.63. Our results showed that 76.1 and 76.8 % of the ROH segments were short (<5 Mb) for the LA and LW breeds, respectively, with an average length of 4.79 Mb for LA and 4.55 Mb in LW. Our estimates of *F*_ROH_ included predominantly short fragments, as mentioned above, which could explain the low correlation between *F*_x_ and *F*_ROH_ that we observed.

Although we found low correlations between inbreeding estimates using genomic and pedigree data, we did observe higher levels of homozygosity in the LW than in the LA breeds with all three methods. Both lines have been selected for several years but the LW breed was imported eight years earlier than the LA breed. Since 2006, selection pressure has increased in both lines, and mating between close relatives is avoided to minimize the rate of inbreeding per generation. The number of animals maintained in the herd was larger for the LA breed than for the LW breed and selection intensity was stronger on the LA than on the LW population but lower levels of homozygosity were observed for the LA population. This could be due to the larger average *N*_*e*_ maintained during the past generations for LA than for LW (Fig. 3) or to lower coancestry between the individuals selected as breeders.

Pedersen et al. [45] and Sonesson et al. [16] proposed the inclusion of genomic inbreeding information for GS, because estimation of inbreeding, based on pedigree information only, underestimates the levels of inbreeding and does not consider unaccounted parentage errors that can accumulate through generations. This is in agreement with our findings, which indicate the need to reconsider the weaknesses that are associated with estimates of population diversity that are based on pedigree information only.

## Conclusions

The use of genomic data to evaluate population diversity in livestock animals can provide new and more precise insights into the effects of intense selection on production traits. Resulting information and knowledge can be used to effectively increase response to selection by appropriately managing the rate of inbreeding, minimizing negative effects from inbreeding depression, and maintaining desirable levels of genetic diversity. For populations with a low level of inbreeding, the use of genomic information has greatly increased the accuracy of genetic diversity estimates. Therefore, major short- and long-term positive impacts of selection response are expected as genomic data is widely incorporated into commercial or cooperative-based breeding programs of all sizes.
